# Gene expression profiling impacts treatment decision making in newly diagnosed multiple myeloma patients in the prospective PROMMIS trial

**DOI:** 10.1002/jha2.209

**Published:** 2021-05-11

**Authors:** Noa Biran, Binod Dhakal, Suzanne Lentzsch, David Siegel, Saad Z. Usmani, Adriana Rossi, Cara Rosenbaum, Divaya Bhutani, David H. Vesole, Cesar Rodriguez, Ajay K. Nooka, Frits van Rhee, Lisette Stork‐Sloots, Femke de Snoo, Pritish K. Bhattacharyya, Durga Prasad Dash, Sena Zümrütçü, Martin H. van Vliet, Parameswaran Hari, Ruben Niesvizky

**Affiliations:** ^1^ Myeloma Division John Theurer Cancer Center Hackensack University Medical Center Hackensack New Jersey USA; ^2^ Division of Hematology and Oncology Department of Medicine Medical College of Wisconsin Milwaukee Wisconsin USA; ^3^ Division of Hematology and Oncology Herbert Irving Comprehensive Cancer Center Columbia University New York New York USA; ^4^ Levine Cancer Institute Charlotte North Carolina USA; ^5^ Department of Medical Oncology New York Presbyterian Hospital‐Weill Cornell Medical Center Weill Cornell Medicine New York New York USA; ^6^ Multiple Myeloma Program Lombardi Comprehensive Cancer Center & Medstar Georgetown University Hospital Georgetown University Washington District of Columbia USA; ^7^ Wake Forest Baptist Comprehensive Cancer Center Winston‐Salem North Carolina USA; ^8^ Winship Cancer Institute Emory University Atlanta Georgia USA; ^9^ Myeloma Center University of Arkansas of Medical Sciences Little Rock Arkansas USA; ^10^ Medex15 Amsterdam The Netherlands; ^11^ Versiti Blood Center of Wisconsin Milwaukee Wisconsin USA; ^12^ SkylineDx Rotterdam The Netherlands

**Keywords:** clinical trials, gene arrays, gene expression, multiple myeloma

## Abstract

Multiple myeloma (MM) is a heterogeneous hematologic malignancy associated with several risk factors including genetic aberrations which impact disease response and survival. Thorough risk classification is essential to select the best clinical strategy to optimize outcomes. The SKY92 molecular signature classifies patients as standard‐ or high‐risk for progression. The PRospective Observational Multiple Myeloma Impact Study (PROMMIS; NCT02911571) measures impact of SKY92 on risk classification and treatment plan. Newly diagnosed MM patients had bone marrow aspirates analyzed for SKY92. Physicians completed a questionnaire for each patient capturing risk classification, hypothetical treatment plan, and physician confidence in the treatment plan, before and after unblinding SKY92. One hundred forty seven MM patients were enrolled. Before unblinding SKY92, physicians regarded 74 (50%) patients as clinical standard‐risk. After unblinding SKY92, 16 patients were re‐assigned as high‐risk by the physician, and for 15 of them treatment strategy was impacted, resulting in an escalated treatment plan. For the 73 (50%) clinical high‐risk patients, SKY92 indicated 46 patients to be standard‐risk; for 31 of these patients the treatment strategy was impacted consistent with a de‐escalation of risk. Overall, SKY92 impacted treatment decisions in 37% of patients (*p* < 0.001). For clinical decision‐making, physicians incorporated SKY92, and the final assigned clinical risk was in line with SKY92 for 89% of patients. Furthermore, SKY92 significantly increased the confidence of the physicians’ treatment decisions (*p* < 0.001). This study shows potential added value of SKY92 in MM for treatment decision making.

## 1 INTRODUCTION

Multiple myeloma (MM) is a hematological cancer, characterized by accumulation of clonal plasma cells in the bone marrow, leading to impairment of hematopoiesis and the production of monoclonal immunoglobulin. It accounts for 1.8% of all cancers with an estimated 32, 270 newly diagnosed cases and 12, 830 deaths for 2020 in the US, and in Europe >48,000 new cases and 31,000 deaths [1, 2]. MM is a very complex heterogenous disease that changes genetically at each relapse in an individual patient. The median overall survival has improved toward 4–10 years [3].

To combat the heterogeneity of the disease, a rapidly increasing number of new therapies have been introduced to the clinical landscape [4]. These drugs are used in single‐agent, doublet, triplet, and quadruplet regimens, and physicians often incorporate autologous or allogeneic stem cell transplant in eligible patients [5]. With the development of novel agents and various combinations, treatment decisions have become increasingly complex.

In order to navigate this complex clinical landscape, guidelines such as the International Myeloma Working Group (IMWG) and Stratification for Myeloma and Risk‐Adopted Therapy (mSMART) recognize patient risk classification as an important tool [5, 6]. Risk classification provides information based on disease biology to report on prognosis that is often used to guide therapeutic decisions. Some risk classifications are based solely on clinical parameters (e.g., International Staging System) [7], while others include cytogenetic aberrations (Revised‐International Staging System [R‐ISS], mSMART) [6, 8]. The National Comprehensive Cancer Network (NCCN) panel agrees that gene expression profiling (GEP) is "a useful tool, that may be helpful to estimate aggressiveness of the disease, helping to make rational therapeutic decisions and individualize treatment" [9]. Although the recently published European Hematology Association‐European Society of Medical Oncology (EHA‐ESMO) clinical practice guidelines remain more conservative and state that “no prognostic factor or staging system, including R‐ISS or GEP, is used routinely to define risk‐adapted strategy” [10].

The SKY92 algorithm is a prognostic, 92‐gene expression signature, also known as EMC‐92. It provides a binary classification, standard risk or high‐risk for disease relapse, [11] and improves the prediction of prognosis for MM patients in clinical practice [12]. SKY92 has been analytically and clinically validated and demonstrated both prognostic accuracy for overall and progression free survival in newly diagnosed and relapsed setting as well as independence of other risk stratification markers in multivariate analyses (Table 1) [11, 12, 13, 14, 15, 16, 17, 18, 19, 20, 21, 22, 23]. This prospective clinical utility study assessed the impact of SKY92 for newly diagnosed MM on risk stratification and treatment decisions and their confidence in the recommended treatment plan.

**TABLE 1:** SKY92 clinical validation studies

Cohort	MM type*	*N*	SKY92 high‐risk (%)	Hazard ratio OS (*p*‐value)	Hazard ratio PFS (*p*‐value)
HOVON‐65/GMMG‐HD4^11^	ND	329	–		
TT2 [11]	ND	351	68 (19%)	3.4 (<0·0001)	
APEX [11]	RR	264	43 (16%)	3.0 (<0·0001)	1·7 (0.0058)
TT3 [12]	ND	254	47 (19%)	4.5 (<0·0001)	
MMGI [13]	ND	91	19 (21%)	8.2 (<0·0001)	
GIMEMA‐MMY‐3006 VTD [14]	ND	114	23 (20%)	4.0 (0·0037)	
CoMMpass [15]	ND	632	116 (18%)	3.1 (<0·0001)	
HOVON‐87/NMSG‐18^16^	ND	190	26 (14%)	2.6 (<0·0001)	2.4 (<0.0001)
KRd trial [17]	ND	16	5 (31%)		8.2 (0.017)
CarThaDex trial [18]	ND	20	5 (25%)		2.8 (0.12)
EMN‐02/HOVON‐95^19^	ND	179	36 (20%)		
E‐MTAB‐1038 [20]	ND/RR	66	13 (20%)	2.6 (0·044)	
TT6 [20]	RR	55	11 (20)	10.3 (0·00015)	
MMpredict non‐trial set [21]	ND/RR	155	34 (22%)	4.5 (<0·0001)	2.7 (<0.0001)
MUKseven trial [22]	RR	48	9 (25%)		2.9 (0.037)
MRC‐IX [23]	ND	246	51 (21%)	2.2 (<0·0001)	
MRC‐XI [23]	ND	329	81 (25%)	3.9 (<0·0001)	2.6 (<0.0001)
Total		**3.339**	**587**		

* ND = newly diagnosed; RR = relapsed/refractory.

## 2 PATIENTS AND METHODS

### 2.1 Study design

**FIGURE 1:**
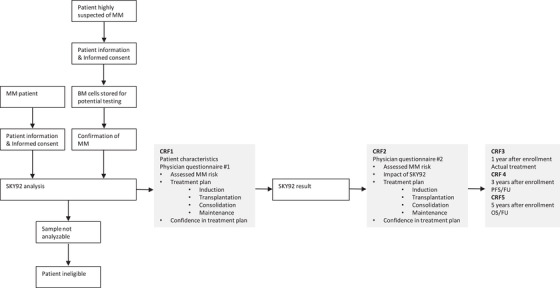
Study design

This study is an observational, prospective, multi‐center study to assess the impact of SKY92 (SkylineDx, the Netherlands) test results on physician decision making regarding risk classification and treatment plan in newly diagnosed MM patients combined with the physician's confidence in the chosen treatment plan (study design: Figure 1). Inclusion criteria were age of at least 18‐year‐old; MM according to the IMWG criteria; candidate for systemic therapy that includes an IMiD and/or proteasome inhibitor; no more than 8 weeks of first line therapy for MM; and a signed informed consent. Exclusion criteria were an Eastern Cooperative Oncology Group performance status over 3 and a bone marrow sample that failed quality criteria for SKY92 testing. The study was approved by institutional review boards of participating centers. The study protocol (PROMMIS) was registered in the clinicaltrial.gov database (NCT02911571). Part of the diagnostic bone marrow sample was analyzed for SKY92 in the local reference laboratories (Hackensack University Medical Center, Columbia University Medical Center, Versiti Blood Center of Wisconsin, and Carolinas Pathology Group) or in SkylineDx’ CAP/CLIA laboratory in San Diego.

The treating physician completed a questionnaire for each patient prior to unblinding the SKY92 risk result, which assessed their MM risk for progression based on their routine clinical practice (*Standard or High‐Risk*), their proposed treatment plan, and their confidence in the proposed treatment plan. After unblinding the SKY92 result, another questionnaire was completed repeating the same questions and the following additional question and answers measuring the impact of SKY92 on the treatment plan: *Did MMprofiler SKY92 impact your treatment intention for this patient? (Yes; my patient will now be treated as High/Standard Risk while prior to MMprofiler SKY92 I considered my patient's myeloma Standard/High Risk; Yes; it was helpful because it confirmed my treatment strategy; No; I considered my patient's myeloma High/Standard Risk prior to MMprofiler SKY92 and also MMprofiler SKY92 is High/Standard Risk; Other, please specify*). Electronic clinical report forms were completed for each patient capturing clinical and pathological characteristics.

### 2.2 SKY92

Fresh bone marrow aspirates were collected in heparin or ethylenediaminetetraacetic acid (EDTA) containing tubes and processed using a Ficoll density gradient, followed by immunomagnetic separation of CD138 positive plasma cells. After RNA extraction, quantity, purity, and integrity were measured. cDNA was prepared, and fragmented cRNA was combined with hybridization reagents to produce hybridization cocktails. These cocktails were hybridized to a SKY92 microarray (U133 Plus 2.0 GeneChip, Thermo Fisher) and scanned on a microarray platform, GCS3000Dx2. If all 10 quality control acceptance criteria were met, the SKY92 score was calculated [11].

### 2.3 End points

The primary end point was the percentage of patients for whom SKY92 led to an alteration in the treatment plan (defined as the hypothetical treatment if the physician could use the SKY92 result). A secondary end point was the physician's confidence in their treatment decisions.

### 2.4 Statistical analysis

We analyzed the proportion of patients whose treatment plan changed after unblinding SKY92, using a two‐sided Exact Binomial test. The principal investigators were aligned considering 15% to be the acceptable threshold of clinical relevance. A comparison of the physician's confidence in the treatment plan before and after unblinding SKY92 results was performed using the Exact test for symmetry, with post hoc testing for differences between pairs of confidence categories, adjusting for multiple testing by the Holm‐Bonferroni method. Clinical and pathological characteristics and risk distribution of SKY92 are summarized. IMWG risk stratification was defined as low risk with ISS I/II, absence of any of t(4;14), del(17p), and gain(1q), and age under 55, high risk with ISS II/III and t(4;14) or del(17p), and standard risk are all other patients [5]. R‐ISS was defined as stage I are those with serum β2‐microglobulin < 3.5 mg/L, serum albumin ≥ 3.5 g/dl, absence of any of del(17p), t(4;14), t(14;16), and normal LDH; stage III are those with serum β2‐microglobulin ≥ 5.5 mg/L, and either presence of del(17p), t(4;14), t(14;16), or high LDH, stage II are those not classified as stage I or III [8].

## 3 RESULTS

### 3.1 Patients

**FIGURE 2:**
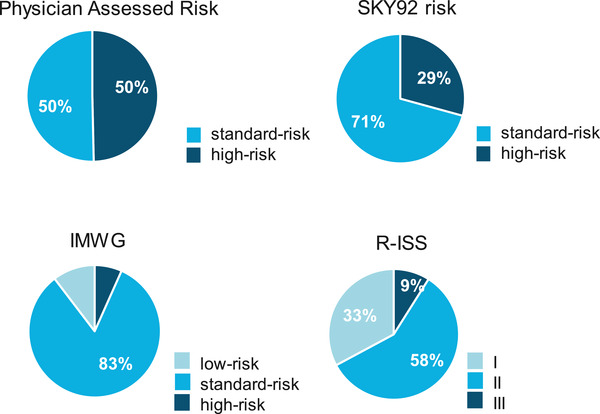
Pie charts of risk assessment by physician before unblinding of SKY92, SKY92, R‐ISS, and IMWG. Pie charts depicting risk distribution for physician assessed risk in standard‐risk and high‐risk categories prior to unblinding SKY92, risk distribution for patients according to SKY92 categories standard‐risk and high‐risk, as well as according to R‐ISS categories I, II, and III (unknown for 14 patients), and IMWG categories low, standard, and high‐risk (unknown for 12 patients)

Two‐hundred fifty patients signed informed consent between February 2018 and April 2020 in nine participating institutions. One hundred three patients were screen failures because of no IMWG “active” MM diagnosis (*n *= 32), bone marrow sample quality not sufficient for SKY92 analysis (*n *= 54) and other reasons, for instance bone marrow sample could not be collected or patients withdrew consent (*n *= 17). One hundred forty‐seven patients were enrolled, and 30 physicians (hemato‐oncologists) completed the questionnaires. Clinicopathologic characteristics of the patients are summarized in Table 2. The median age was 66 years (range 35–86), and 63% were male. A total of 29% (43 of 147) patients were SKY92 high‐risk (Figure 2). The risk distribution by R‐ISS was 33% (44 out of 133) stage I, 58% (77 out of 133) stage II, 9% (12 out of 133) stage III and by IMWG: 10% (14 of 135) low​‐risk, 83% (112 of 135) standard‐risk, and 7% (9 of 135) high‐risk. For one patient, cytogenetics were not assessed. The following number of patients had high‐risk cytogenetic features (locally assessed): del(17p) in 15 of 146 (10%), gain(1q) in 64 of 146 (44%), t(4;14) in nine of 146 (6%), and t(14;16) in five of 146 (3%) patients.

**TABLE 2:** Patient characteristics according to SKY92 risk groups

	SKY92 Standard‐risk (*n *= 104)	SKY92 High‐risk (*n *= 43)	Total (*n *= 147)
**Age, median (range)**	63 (35–85)	68 (36–86)	66 (35–86)
**Gender (% male)**	63 (61%)	29 (67%)	92 (63%)
**β2‐microglobulin ≥ 3**·**5** **mg/L**	47/96 (49%)	26/41 (63%)	73/137 (53%)
**Albumin < 3**·**5** **g/dL**	30/103 (29%)	21/42 (50%)	51/145 (35%)
**LDH ≥ upper limit of normal**	12/102(12%)	11/39 (28%)	23/141 (16%)
**IMWG low‐risk**	13/95 (14%)	1/40 (2%)	14/135 (10%)
**IMWG standard‐risk**	80/95 (84%)	32/40 (80%)	112/135 (83%)
**IMWG high‐risk**	2/95 (2%)	7/40 (18%)	9/135 (7%)
**R‐ISS I**	38/95 (40%)	6/38 (16%)	44/133 (33%)
**R‐ISS II**	53/95 (56%)	24/38 (63%)	77/133 (58%)
**R‐ISS III**	4/95 (4%)	8/38 (21%)	12/133 (9%)
**Del (17p)**	11/103 (11%)	4/43 (9%)	15/146 (10%)
**Gain (1q)**	34/103 (33%)	30/43 (70%)	64/146 (44%)
**t (4;14)**	3/103 (3%)	6/43 (14%)	9/146 (6%)
**t (14;16)**	3/103 (3%)	2/43 (5%)	5/146 (3%)

### 3.2 Physician's change in risk classification

**FIGURE 3:**
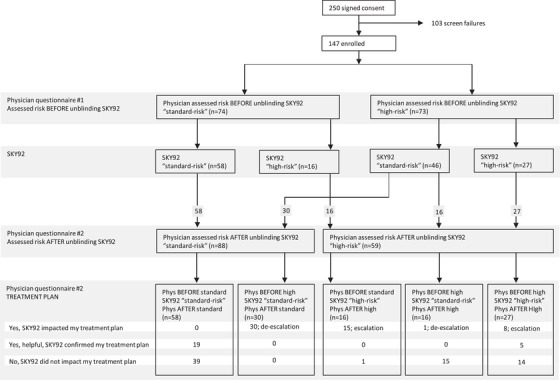
Flow diagram of all patients

**FIGURE 4:**
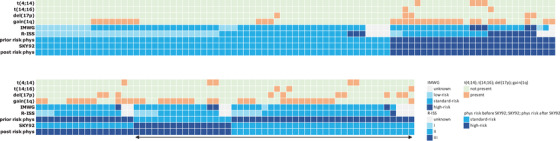
Heatmap of cytogenetic abnormalities and risk classification. Figure depicting comparison of cytogenetic abnormalities and risk classification for each of the *n *= 147 patients included in this analysis. Each column represents a patient, each row represents a cytogenetic abnormality (either present or not present), and risk classification system. For each patient, risk according to IMWG and R‐ISS is depicted. The seventh row is the physician assessed risk prior to unblinding SKY92 (“prior risk phys”); the eighth row is the risk as determined by SKY92. The last row is the physician assessed risk after unblinding SKY92 (“post risk phys”). The *n *= 46 patients for which physicians changed their risk assignment are highlighted by the arrow

Prior to unblinding the SKY92 result, physicians assessed the patient's risk classification according to their own clinical routinely used methods. On the basis of that assessment, the physician is asked to summarize the patient's risk classification into either *standard* or *high‐risk*. In 73 of 147 (50%) of patient cases, the physician determined the patient as having clinically high‐risk MM. Consequently, the other half (74 of 147) of patient cases were assessed standard‐risk. After unblinding the SKY92 result, physicians determined the final risk classification for each patient. In their final assessment they indicated 59 of 147 (40%) of patients to be clinically high‐risk and subsequently 88 of 147 (60%) of patients to be standard‐risk. All the patients that received a SKY92 high‐risk result were considered high‐risk by the physicians in their final assessment. This means that 16 patients, previously considered standard‐risk, were now perceived to be high‐risk classification on the basis of SKY92. A total of 30 patients, previously considered high‐risk, were de‐escalated in physician estimation to a standard‐risk classification on the basis of SKY92. These changes in perceived risk were reported by physicians as impacting proposed treatment plans especially post auto transplant options. The impact of SKY92 on the physicians’ clinical risk classification is captured in Table 3A (Figure 3 for a flow diagram depicting risk and treatment plan distribution in the study). For 131 of 147 patients (89%), the final risk classification coincides with the SKY92 result. More specifically, all the patients classified standard risk in the final assessment and had a standard‐risk SKY92 outcome (100% concordance). The concordance was 73% for the high‐risk cases (43 of 59) (Table 3B). A comparison of cytogenetic abnormalities and risk classification per patient and impact on risk assessment is depicted in Figure 4.

### 3.3 Physician's change in hypothetical treatment plan

The physician's change in risk classification after unblinding the SKY92 result resulted in a change in the proposed hypothetical treatment plan in several patient cases. For 31 of 46 (67%) patients that were regarded clinically high‐risk by the physician prior to unblinding SKY92, the treatment plan was impacted, consistent with a downgrading of risk, and resulted in a de‐escalated treatment plan when SKY92 reported a standard‐risk result (Figure 3). Furthermore, for 15 of 16 (94%) patients assessed as clinically standard‐risk by the physician prior to unblinding SKY92, the treatment plan was impacted resulting in an escalated treatment plan when SKY92 reported a high‐risk result. Finally, there were eight clinically high‐risk patients prior to unblinding SKY92, where the physician still escalated the treatment plan because SKY92 was also high‐risk. For 19 concordant standard‐risk patients and five concordant high‐risk patients, the physician indicated the SKY92 test to be helpful because it confirmed their treatment plan. In summary, treatment plan decisions were impacted by SKY92 in 37% (54 of 147) patient cases, which is significantly different from the predefined threshold for clinical relevance of 15% (*p *< 0.001).

### 3.4 Physician's confidence in treatment plan

One of this study's secondary objectives was to assess the physician's confidence in their treatment plan. Before unblinding SKY92, physicians indicated they were not at all confident (*n *= 5, 3%), not confident (*n *= 8, 5%), ambivalent (*n *= 18, 12%), confident (*n *= 96, 65%), and strongly confident (*n *= 20, 14%) about their treatment plan (Table 4). After unblinding SKY92, the physician's confidence changed to ambivalent (*n *= 6, 4%), confident (*n *= 80, 54%), and strongly confident (*n *= 61, 41%) about their treatment plan. The effect is most prominent in the shift from confident before, to strongly confident after receiving the SKY92 test result (*n *= 34, 23% patients, *p *< 0·001). Overall, utilizing SKY92 led to physicians having significantly more confidence in their treatment plan (*p *< 0.001).

**TABLE 4:** Physician's confidence in their treatment plan before and after unblinding of SKY92 results

	After unblinding of SKY92					
Before unblinding of SKY92	Not at all confident	Not confident	Ambivalent	Confident	Strongly confident	Total
Not at all confident	0	0	0	5	0	**5**
Not confident	0	0	1	3	4	**8**
Ambivalent	0	0	4	10	4	**18**
Confident	0	0	1	61	34	**96**
Strongly confident	0	0	0	1	19	**20**
Total	**0**	**0**	**6**	**80**	**61**	**147**

**TABLE 3A:** Clinical risk before and after unblinding SKY92

	Risk assignment **after unblinding** SKY92		
Risk assignment **before** unblinding SKY92	Standard‐risk	High‐risk	Total
Standard‐risk	**58**	16	74 (50%)
High‐risk	30	**43**	73 (50%)
Total	88 (60%)	59 (40%)	147 (100%)

**TABLE 3B:** Clinical risk after unblinding SKY92 compared with SKY92

	SKY92			
Clinical risk assignment after unblinding SKY92	Standard‐risk (*n *= 104; 71%)	High‐risk (*n *= 43; 29%)	Final assigned clinical risk in concordance with SKY92*	
standard‐risk (*n *= 88; 60%)	**88**	0	100%	Total: 89%
high‐risk (*n *= 59; 40%)	16	**43**	73%	

* Percentage of patients in whom the clinical risk after unblinding of SKY92 was in line with the SKY92 test result (i.e., clinical standard‐risk patients with a standard‐risk SKY92 profile and clinical high‐risk in patients with a high‐risk profile).

### 3.5 Sub‐analysis 65 years and older

A sub‐analysis was done in the eligible Medicare beneficiary population. For the 75 patients aged 65 years and older, an overall concordance of 87% was seen between the physician's final risk assessment after unblinding SKY92 and the SKY92 test result (supplemental figure). SKY92 had an overall impact of 27% on the physician's treatment plan which is significantly different from the predefined threshold of clinical relevance of 15% (*p *= 0.009).

## 4 DISCUSSION

The definition for high‐risk MM is recognized to be constantly evolving with advances in diagnostics and therapeutics [24]. Many factors are being used to determine whether a patient should be regarded high‐risk for disease progression and hence be treated more rigorously. The current study is not intended to show SKY92 improves outcome, since this has already been validated in previous studies [11, 12, 13, 14, 15, 16, 17, 18, 19, 20, 21, 22, 23]. The study is aimed to assess whether SKY92 could provide additional prognostic guidance for physicians. The results from this first prospective multicenter study show the potential utility of the robust gene expression‐based risk classifier SKY92 in providing additional guidance for risk assessment.

Unblinding SKY92 results led to a renewed clinical risk assessment by the investigators and an optimized, adapted treatment plan in 37% of patients. In 46 MM patients, initially deemed to be high‐risk by the physician, treatment was de‐escalated in 30 cases (65%) after patients were reclassified to standard risk by the physician based on the SKY92 result. Conversely, there were 16 (22%) patients who were initially classified as standard risk but were reclassified to high‐risk based on the SKY92 high‐risk result, and in 15 cases (94%), treatment was intensified. The final clinical risk assessment was in line with SKY92 in 89% of patients. Furthermore, knowing the SKY92 result led to an increased confidence of the physician in their proposed treatment plan. This increased confidence is of paramount importance to the complex heterogeneous clinical practice of MM in which physicians are confronted with a large, evolving body of data, and its significance in how to treat a patient.

Overall, the PROMMIS study population seems to be fairly representative as far as risk classification when compared with the pooled dataset used for R‐ISS development, [8] where 62% of patients were in the intermediate‐risk group (52% in PROMMIS), 28% were in the low‐risk group (30% in PROMMIS), and 10% were in the high‐risk group (8% in PROMMIS). The higher number of high‐risk patients by means of SKY92 (29%) compared to clinical risk classification guidelines such as R‐ISS, is a known phenomenon, [16, 23] suggesting that a substantial number of high‐risk patients cannot be diagnosed by current clinical methods alone.

With 29% of patients SKY92 high‐risk in this cohort (compared to 15–25% of high‐risk patients in historical, newly diagnosed cohorts [11, 12, 13, 14, 15, 16, 17, 18, 19, 20, 21, 22, 23]), it seems reasonable to surmise that physicians selected higher risk patients to be included in the PROMMIS study. This notion is reflected by the observation that 50% of patients were thought to have high‐risk myeloma by their physicians based on their routine clinical practice before unblinding of the SKY92 result, this may include but is not restricted to standard risk‐stratification methods like R‐ISS or cytogenetic aberrations such as amplification or duplication of chromosome 1q or other translocations/copy‐number abnormalities that have adverse prognostic implications [25, 26]. Other parameters could have been derived from modern imaging techniques such as MRI and CT‐PET scanning; [27, 28, 29] patient‐specific high‐risk features such as old age, poor performance status, and comorbidities; or clinical features such as primary plasma cell leukemia and extramedullary disease [30]. It is interesting to note that abnormalities of 1q occurred in 70% of SKY92 high‐risk patients compared to 33% in SKY92 standard‐risk patients. The SKY92 signature is enriched for genes located on 1q [11], and similar findings have been found for other risk scores based on GEP such as UAMS70 [31]. Despite some overlap in 1q gene enrichment, SKY92 seems to identify a larger group of patients as high‐risk (15%–25%) compared with the UAMS70 risk score, which identifies 12% of patients as high‐risk [31]. Two papers [12, 23] are available with comparative data for both risk scores: in a pooled dataset both GEP profiles were analyzed and showed that UAMS70 identified 9% of patients as high‐risk whereas SKY92 identified 18% of patients as high‐risk [12]. In the Myeloma XI trial, a comparative analysis for quantitative risk scores score was performed showing a significant correlation (79%) between the two profiles. SKY92 high‐risk patients had significantly shorter PFS and OS compared with their SKY92 standard‐risk counterparts. Similar performance of prognostication was shown for UAMS70 on OS, but not PFS [23]. Other techniques for molecular profiling of MM such as next generation sequencing, DNAseq or RNAseq, have not been widely adopted in MM (yet) and are currently mostly used for detection of measurable residual disease [32].

One limitation in this study is the screen failure rate of 103 patients. SKY92 is developed and validated for “active” MM. Upon bone marrow sample collection for SKY92 assessment, it was not always clear whether the diagnosis was symptomatic versus smoldering myeloma, leading to screen failures. Once the test is validated for smoldering myeloma, the screen failure rate could be reduced. Nevertheless, the bone marrow sample quality was not sufficient for SKY92 analysis for 54 patients, which might be prevented by more stringent instructions for obtaining aspirate.

The present study shows that SKY92 provides alternative risk classification beyond currently used routine clinical methods like R‐ISS and adverse fluorescence in situ hybridization (FISH) such as deletion (17p), t(4;14), and t(14;16). There were 29 patients with these adverse FISH criteria, and only 12 (41%) were high‐risk by SKY92. These numbers are in line with the retrospective analysis of SKY92 in the Myeloma XI trial which showed a similar percentage of patients with del(17p), t(4;14), and t(14;16) abnormalities classified as SKY92 high‐risk [23]. Patients in the Myeloma XI trial were followed for 72 months and demonstrated SKY92 to be a better prognostic biomarker. Also, this study showed that SKY92 high‐risk patients are unlikely to benefit from single agent lenalidomide maintenance therapy, and in such patients intensified therapy with combination agents may be beneficial [23]. Currently used clinical risk assessment models are suboptimal, and this study demonstrates that SKY92 provided additional information and potentially impacts clinical decision making. Physicians aligned their assigned risk closely with SKY92 results as opposed to other risk stratification systems, indicating the potential added value. There was improved physician confidence in treatment decisions after SKY92 results in a disease where selecting therapy is of paramount importance.

## CONFLICT OF INTEREST

Binod Dhakal has served on the advisory board of Takeda, Amgen, and Jansen and has received honorarium from Celgene. Suzanne Lentzsch reports equity ownership and membership on an entity's board of directors or advisory committees for Caelum Biosciences, consultancy for Bayer, Janssen, Takeda, BMS, Proclara, Abbvie, speakers bureau for Clinical Care Options, consultancy and research funding for Sanofi, honoraria from MMRF and IMF, research funding from Karyopharm and patents and royalties for 11‐1F4mAb as anti‐myeloid strategy for Columbia University. David Siegel reports membership on an entity's board of directors or advisory committees, research funding, and speaker's bureau for Takeda, Amgen, Celgene, BMS, Janssen, Celularity, and Karyopharm. Saad Z. Usmani reports research funding, speakers bureau activities, and consultancy for Amgen, Array Biopharma, BMS, Celgene, Janssen, Merck, Pharmacyclics, Sanofi, Takeda and SkylineDx. Adriana Rossi reports consultancy for Janssen, Amgen, BMS, and research support from BMS. David H. Vesole reports speaker's bureau activities for Takeda, Amgen, BMS, Janssen, GSK. Ajay K. Nooka has served on the advisory board and received honoraria from Amgen, BMS/Celgene, Takeda, Janssen, GSK, Karyopharm, Oncopeptides, Spectrum Pharmaceuticals , and Adaptive Technologies. Parameswaran Hari reports research funding, honoraria and consultancy for Celgene, Takeda, BMS, Janssen, Kite, Amgen, Spectrum, and Sanofi. Divaya Bhutani reports an entity's board of directors or advisory committees for Sanofi. Ruben Niesvizky reports consultancy and research funding for Takeda, Amgen, BMS, Janssen, and Celgene. Lisette Stork‐Sloots and Femke de Snoo are consultants for SkylineDx. Sena Zümrütçü and Martin H. van Vliet are employees and stockholders of SkylineDx. The remaining authors declare no competing financial interests.

## AUTHOR CONTRIBUTIONS

Suzanne Lentzsch, David Siegel, Saad Z. Usmani, Lisette Stork‐Sloots, Femke de Snoo, Parameswaran Hari, Ruben Niesvizky were responsible for the study design. Noa Biran, Binod Dhakal, Suzanne Lentzsch, David Siegel, Saad Z. Usmani, Adriana Rossi, Cara Rosenbaum, Divaya Bhutani, David H. Vesole, Cesar Rodriguez, Ajay K. Nooka, Frits van Rhee, Parameswaran Hari, and Ruben Niesvizky performed the research. Pritish K. Bhattacharyya and D.P. Dash performed SKY92 analysis. Sena Zümrütçü critically reviewed all QC data and provided technical assistance to the sites. Lisette Stork‐Sloots and Femke de Snoo analyzed and interpreted the data and wrote the manuscript. All authors participated in data interpretation and critical appraisal of the manuscript and approved the final manuscript.

## Data Availability

The data that support the findings of this study are available on request from the corresponding author. The data are not publicly available due to privacy or ethical restrictions.
